# Lost in translation? Jáchym Topol’s *The Devil’s Workshop* and its local and transnational reader

**DOI:** 10.12688/openreseurope.17816.1

**Published:** 2024-08-16

**Authors:** Bernadette Ščasná

**Affiliations:** 1 School of Humanities, Tallinn University, Tallinn, Harju County, 10120, Estonia

**Keywords:** cultural memory, memorialization efforts, difficult heritage, transnational literature, reception study, Terezín concentration camp, Khatyn massacre

## Abstract

Jáchym Topol, who is a Czech writer and poet, has several times emphasized that he does not care about his readers, especially foreign ones who get to read translated versions of his work, as it is not his job to be understood. With the rise of transnational WWII-related literature in the Czech Republic in the last two decades, I explore how his novel *The Devil’s Workshop* has become an important work in the realm of transnational literature, despite Topol’s peculiar writing style and stance towards his international readers. In my analysis, I explore Topol’s writing style, the novel’s socio-historical context through the concept of difficult heritage and the juxtaposition of Czech and Belarusian WWII narratives, as well as the novel’s local and international reception.

## Plain language summary

This article’s aim is to explore how writing style, literary aesthetics, and content can influence international and local reception of a novel taking place in a transnational setting. I focus on the context and reception of a novel called *The Devil’s Workshop,* written by a Czech author Jáchym Topol, first published in Czech in 2009. Furthermore, I also explore the novel’s place within transnational literature. Transnational literature can help bridge the gap between different countries’ memory and heritage, and that is what Topol’s novel does in regards to Czech and Belarusian WWII–related past. The context of the novel is explored through concepts including “difficult heritage”, “memory entrepreneurship”, and “genocide tourism”. In terms of the context analysis, this article focuses on the juxtaposition of WWII-related memorialization practices in the former Czech concentration camp Terezín and a Belarusian massacre site in Khatyn, which lie at the core of the novel. The novel’s reception is explored on both local and international level, through publically available online reviews. In terms of reception, I focus on the reader’s interpretations of the novel, their stance towards its ideas, as well as Topol’s writing style and use of dark humor. The results show how a novel written for a local audience managed to open up the floor for self-reflection and raise awareness of the past and WWII-related memory differences within Europe.

## Introduction

I really don’t care about the person whom you name ‘an English reader’ or a ‘foreign reader’, it is not my job…it is the job of a journalist or a commentator to be understood anywhere. I write what I feel and what I want and what I need to write and that is all I care about. I am not interested in writing a Euroburger and I don’t really need everyone to get what I write. I am not dependent on my income from a book, I can support my family by different means and writing is my freedom. ( Topol, 2010a)

This article analyzes reception of the novel *The Devil’s Workshop* ( Topol, 2013) ^1^ by an eminent Czech writer and poet Jáchym Topol that deals with the memorialization and difficult heritage of the Holocaust in Eastern, Central, and Western Europe and with the massacre of non-Jewish citizens of the Belarusian village of Khatyn by the Nazis. The first part of the novel is set in the Czech Republic on the site of a former Nazi concentration camp, Terezín, and the second part in Minsk, the capital of Belarus, and at the nearby site of the Khatyn massacre ^2^ which is situated about 50 kilometers from Minsk. At the core of *The Devil’s Workshop* lie many juxtapositions that compare memorialization practices of the Holocaust and the Khatyn massacre and commercialization of their memory. In the second part of the novel, the story generates suspense by focusing on a group of Belarusians who wish to break free from the memory suppression in their country and adapt to the standards present in what they consider to be the “Western Europe” and commercialize their dark heritage for profit. To reach their goal, they invite the novel’s narrator, a Czech man born in the garrison town Terezín, who in the first part of the novel works on a revitalization project in the Czech Republic as a consultant.

The novel cleverly intertwines fact and fiction together with several theoretical ideas about memory and commemoration of atrocities that are familiar from many debates in memory studies. The novel touches upon all these issues on the level of transnational memory. The ideas are incorporated playfully and twisted at their core through the use of the grotesque, satire, and traditional Czech dark humor. Therefore, in my analysis, I will also focus on the aesthetics of the text and on the relationships between the writer, the translator and the readers on a transnational level of literary process.

My opening quote by Jáchym Topol (2010a) is the answer he gave in an interview with the *Prague Monitor* to the question whether he was worried that a foreign reader would not understand his book *Gargling with Tar* ( Topol, 2010b) when it was translated into English and several other languages. The reason why the interviewer asked this question was due to the characteristics of Topol’s writing style that is seen to be mainly catered to local audiences ^3^, and may not be well comprehended or understood by foreign readers. What I mean by “catered to local audiences” here refers to Topol’s use of colloquial language, subtle references to historical events, as well as embedment of dark humor that is typical for Czech literature ( Holý, 2017, pp. 1–2, 208–221; Němcová Banerjee, 1985, pp. 14–17). Topol has written multiple novels on the topics of history, society, war, and memory. Generally, his fictional works ^4^ revolve around the difficulties of Czech society in coming to terms with the aftermath of totalitarian regimes. *The Devil’s Workshop* deals with the memorialization of the Holocaust in the Czech Republic while taking the story to the transnational ^5^ arena. In *The Devil’s Workshop*, I will scrutinize Topol’s writing style and explore how it works both on the local, as well as on transnational level. The aim of this article is to show how, despite being arguably written for local readers, the novel has become an important work of transnational literature as it offers an opportunity for the readers all around the world to engage in processes that create awareness of dark past events.

I will be guided by the questions posed to Topol: is his writing style an “obstacle” for transnational readers? Does the reading experience of local readers strongly differ from that of the transnational readers? If this is the case, then how? I will seek to answer these questions by combining textual analysis and reception studies. First, I will explore the historical and socio-political themes of the novel which show how it contextualizes memory through the concept of the difficult heritage of World War II in different parts of Europe. In the next step, I will study the novel’s local and transnational reception to see how it has been read both at “home” as well as abroad.

 I will analyze reception by gathering and analyzing online reviews and comments posted to different sites including Amazon, Goodreads, and the Czech domain called Databazeknih.cz. I have decided to focus on vernacular reviews rather than those written by professional critics as I believe they can provide a lot of interesting information in regard to how novels influence all kinds of readers with different backgrounds around the world. The role of digital participatory culture has become much stronger recently and it is clear that literary studies and other disciplines can greatly benefit from them ( Vadde & Majumdar, 2020). Research has shown that informal reader feedback and interaction on online platforms such as Goodreads can be used to assess the impact that a given book has in terms of its educational and cultural value ( Kousha *et al.*, 2017). All the platforms I have selected allow the readers to express and share their opinions, feelings, and thoughts on different books publicly online with other readers. ^6^ The aspects that I am most interested in are the readers’ interpretations of the novel, their stance towards its ideas, and their thoughts on the aesthetic aspect of the novel. In particular, I am interested in their response to the use of dark humor as one of the striking characteristics of the novel as the author and cultural critics have expressed skepticism in respect to whether it can be “well translated” and hence understood by foreign readers.

## *The Devil’s Workshop:* creating awareness of difficult past through a transnational lens

If *The Devil’s Workshop* is arguably written for a Czech readership, how does it portray and thematize both local Czech history and memory as well as the Belarusian one that is foreign to them? And how readable is such text for international readers? As I have briefly mentioned, Topol’s novel focuses on the will of the Belarusians to break free from the memory suppression, adapt to the standards present in what they consider to be the “Western Europe”, and commercialize the sites of atrocity in order to gain transnational recognition and profit. This novel conceptually represents a rich text which tackles several concepts that are prevalent in memory studies debates and twists them at their core through grotesque, and satire. The concepts that the novel tackles work as covert tools that emphasize its transnational scope and can potentially help to raise readers’ awareness of many issues related to sites of dark heritage.

The novel is set in two different locally specific contexts of history and memory – Terezín and Khatyn – but the analysis of contemporary trends and problems of memorialization of this conceptually rich text is transnational in scope. It discusses ideas related to the sites of dark heritage that are common to many places and hence these ideas work as covert tools that turn the representation of Czech and Belarusian contexts into a transnationally relevant text. The transnational quality can also make the local context more approachable to all types of audiences.

The issues Topol’s novel explores can be described by the concept of “difficult heritage” that describes the past that is of current importance but is contested and difficult to reconcile with publicly, and can force its way into the present in unruly ways ( Macdonald, 2009, p. 1). Topol also deals with what has been described as “memory entrepreneurship”, a practice of trying to revise, actively create, and popularize historical memories ( Autry, 2017, p. 27) and “genocide tourism” which involves popularization of travel to the sites of genocide ( Beech, 2009). The role of these ideas within the text will be explained as I move chronologically through the plot and explore the memory-related issues portrayed while focusing on their transnational character.

The story of the novel is told from the perspective of an unnamed narrator who was born and raised in the Czech town Terezín, the site of a former WWII Nazi concentration camp ^7^. The town in the novel is dilapidated and nearly in ruins. Yet, it is inhabited by a group of locals, including the narrator. Terezín is where the narrator in collaboration with the locals begin working on a project dedicated to the revitalization of the town, improving its tourism, and helping its visitors heal the scars that the past has imprinted on them. They establish their headquarters in a building which they name Comenium, inside a building which up until that moment functioned as a squat for those with no place to go. However, the revitalization project and efforts to preserve the site are not officially approved by the government due to the intervention of scholars and board members of an official organization called Monument. The Monument works on state-funded educational trails of war crimes and terror and decides not to preserve the town of Terezín due to lack of finances. The last inhabitants who refuse to move out are the only thing stopping the bulldozers from destroying the town.

The man responsible for the revitalization project is called Uncle Lebo who was born in the Terezín concentration camp. He appoints himself as the lead memory entrepreneur on the site with the goal of protecting the town and keeping it in its original condition. He compiled a list of contacts which included former prisoners of Terezín concentration camp and their relatives and reaches out to them, asking for financial support of the project. However, what Topol at first seemingly represents as an effort to commemorate the victims and protect a site of dark heritage, is in fact a veil covering the more selfish ambitions of the characters, including the narrator – keeping their home:

Every inch of Terezín should exist always and for ever, and… feed the memory of the world. I didn’t care about memory, though. I just needed a place to live. I really hoped Lebo could save the town. And I hoped his contacts could feed us all —everyone living here, even the ones who were only half alive. ( Topol, 2013, p. 29)

However, the narrator is not the only one whose own priorities are elsewhere than in keeping the site of dark heritage alive for the purpose of preserving its dark memory. Uncle Lebo began the project mainly to defend the town from destruction and keep the space for its current inhabitants. He deeply cared for Terezín, and did not wish to see it reduced to a mere monument and educational trails. Thus, with the help of the children and other inhabitants of Terezín, he tried to collect every single stone, bone, and item that reflects its dark history. As the next phase of their plan, they focused on commercialization and commodification of Terezín memory. In hopes of their project’s success, the inhabitants of Terezín capture attention of foreign tourists under the false pretense of memory preservation for the sake of humanity, concealing their own selfish goals:

Rolf s reportage was published with a photograph of the giant Lebo dressed in black, gazing out from the ramparts into the reddening twilight as he declared, *This place of dreadful horror must be preserved for the memory of humanity*. Of course Rolf made up the ‘memory of humanity’ part — Lebo didn’t talk about his activities. And he had no intention of feeding the world’s memory. He just wanted to feed the dying inhabitants of Terezín. ( Topol, 2013, p. 29)

Here Topol represents the possibility of exploiting memory by reaching out to the collective and transnational sphere. By raising awareness of the site and emphasizing the global connectedness of the local memory of Terezín to the transnational memory of the Holocaust, the locals turn to disrespectful practices such as commodification of memory for profit.

The way Topol portrays the Czechs and their disinterest towards their dark history connected to the Holocaust and WWII, partially reflects the actual state of politics of memory in the Czech Republic. While the Czech efforts to reconcile with their dark past related to the Holocaust memory has been fairly successful, the prevalent narratives of memory still have certain problematic issues. The postwar narrative in the Czech part of the communist Czechoslovakia mainly focused on the oppression of the Czechs by the Nazis ( Kalousová, 2022, p. 187). While a lot of progress has been made in challenging this narrative, it is still deeply rooted within Czech memory culture. Czech suffering takes precedence over the Jewish Holocaust (ibid., p. 199). Czech lands were occupied by Germany during WWII and in comparison, to the neighboring Slovakia, which collaborated with Nazis in the deportations of Jews, the Czechs did not collaborate in the Holocaust to the same extent. There were also “no violent group actions such as pogroms” towards Jews from their side ( Sniegon, 20114, p. 94). Thus, the Holocaust was not seen as a problem for the Czech national narrative and was mainly ignored (ibid.).

In contemporary Czech national narrative Jews are usually mentioned in a self-congratulatory manner in the context of the interwar period when Czechoslovakia arguably had a “well-developed democratic culture” which offered generous opportunities for Jewish minorities, and the possibility to officially keep their “Jewish” nationality, like no other Central European country at that time ( Sniegon, 2014, p. 96). It is important to note that while the official discourse of the post-socialist period has focused mainly on Czech suffering, there have been memory actors including historians, writers, and Jewish community leaders who have contested that narrative with a memory that asserts the Jewish Holocaust experience as part of Czech history ( Lichtenstein, 2016, p. 140). While it has remained marginalized, a counter memory of the Holocaust has been shaped to fight the social and political lack of interest in the Holocaust with some degree of success (ibid.). One of the examples of the success is the institutionalization of the annual commemoration of the liquidation of the Terezín family camp in Auschwitz-Birkenau, which took place on 8 March 1944 (ibid.). During the liquidation of the camp, its inhabitants were taken to gas chambers and killed. According to witnesses, during the transportation to the gas chambers, the victims were heard singing the Czech national anthem (Kde domov můj), as well as the Zionist hymn (Hatikvah), among other songs. The presence of the Czech anthem in this context served to emphasize the victims’ duality, encompassing both their Czechness and Jewishness (ibid.). Unfortunately, this is one of many mass murders of Jews, the majority of which has not received enough attention in the Czech Republic, in comparison to non-Jewish ones like the Lidice massacre ^8^ which is part of the Czech victimization narrative (ibid., p.135).

Even the more recent impulses and debates related to the Holocaust in the Czech Republic rarely have come from within its own historical culture. Rather, it has happened as a result of activism, Americanization of culture during the 1990s, or Europeanization efforts for the purpose of joining the EU and NATO ( Sniegon, 2014, p. 207). Thus, Topol’s novel points to an existing lack of interest while creating a sort of an ironic image which questions the reasons for preservation of WWII sites and their memory.

The narrator of Topol’s novel as well as the squatting inhabitants of Terezín display a similar stance towards the Holocaust and remain indifferent to it. The main reason for their work on the revitalization projects stems from the economic advantage it gives them. It is a form of business that helps them keep their homes. They set up what they call the “Amusement Centre” where they sell Kafka T-shirts as souvenirs and a “ghetto pizza” stand for the incoming tourists. Soon they learn that these activities are illegal when they receive indictments from the Monument, claiming that their activities “constituted a gross desecration” of the “sacred site” ( Topol, 2013, p. 51). However, the inconsiderate and careless nature of the project members’ actions is exposed by a visitor from abroad who brings in a different point of view.

Heightened publicity of the project by reportage brings travelers from all around the world to Terezín, and to the Comenium. Among the travelers, is a girl from Sweden called Sara, whose father was a Slovak Jew and whose many relatives died in Terezín. She brings in a Western point of view on Terezín as a site of memory and lives of its locals. She is critical of the fact that the locals have been living on the site of slaughter, selling food there, doing laundry on the exact spot where trains would take prisoners away to extermination camps, and the kids running around the site, playing in morgues. From her Western point of view, where the WWII mass graves are well-maintained and tended to, it is unthinkable how in the Czech Republic – in the East – the people do not seem to care about any of it. She brings up these complaints in her conversation with the narrator. Her point of view brings forth the transnational dimension of the novel, which can encourage the local readers to reflect on the status of Holocaust memory in the Czech Republic and compare it with other countries. It also offers an entry point to the novel and to Terezín for international readers.

Sara, and her presence in Terezín bring out several other significant self-reflections. Her conversation with the narrator invites the readers of the novel to not only reflect upon the issues of commodification, and disrespect towards sites of atrocities or lack of interest in memory, but also upon the role of tourism on the sites of dark heritage. Two types of tourists at such sites are juxtaposed. The narrator compares Sara to other tourists he meets in Terezín:

She was one of the seekers of the bunks, young people with brains darkened by the cloud of terrible past, by the horrors that had befallen their parents, grandparents, relatives, or just by the fact that those horrors had happened at all… Ordinary tourists strolled through Terezín like it was a medieval castle, taking snapshots, shooting videos of the dungeons and torture chambers to show the family afterwards. The bunk seekers would never even think of such a thing. They showed up here crazed with pain, seized by the eternal question every seeker asked: *If it happened here can it happen again*? They knew they weren’t in a medieval castle but in an abyss where the world has been torn apart, a place without mercy or compassion, where anything was possible. ( Topol, 2013, p. 32)

This juxtaposition compares “ordinary tourists” who come to Terezín as to any other tourist attraction, take pictures and shoot videos, as if they were not standing on a site marked by thousands of human deaths to tourists who are seeking answers or a way to heal the scars that the dark past has imprinted on them through transgenerational family trauma. In terms of the second type of tourists, the novel depicts people who are marked by the postmemory ( Hirsch, 2012)) of their ancestors. Hirsh defines postmemory as the challenging relationship between the generation who has personal trauma and memories of WWII and the generation who came after and to whom their experiences were transmitted on a level so deep that it becomes a part of their own personal memory, and thus, the haunting past continues to have an effect in the present ( Hirsch, 2012, p. 5). Comparing the tourists influenced by postmemory to those who see it as a mere tourist attraction creates a striking image that calls for self-reflection by the novel’s readers.

 In recent years, there have been several instances where similar “ordinary” kinds of tourists were targeted in different news articles by Holocaust museums and memorial sites who wished to combat social media disrespect ( Hucal, 2019). Images of smiling tourists with flashy poses in front of the gates of Auschwitz-Birkenau, or photos of people balancing on the train tracks that were bringing in Jewish deportees have drawn attention of the museum staff who have asked for respect for the site on social media (ibid.). Another example is Berlin’s Memorial to the Murdered Jews of Europe whose architectural stones have been used by some as a spot for sunbathing, parkour, or picnic (ibid.). This type of “negative” tourism is yet another significant parallel between the novel and reality. On the one hand, Topol’s critique in the form of satire invites the readers to reflect on their behavior and motivations for visiting such sites. On the other hand, by including tourists who seek answers and healing in his narrative, Topol also emphasizes transnational significance of Holocaust memorials for people from different parts of the world and the importance of paying respect to them. Due to the fact that Topol’s writing is set out to ridicule people’s behavior in the form of satire throughout the novel, it offers a lot of space for self-reflection and opens up space for debates that make one wonder where the limits of memorialization, tourism and commercialization should be.

 The Terezín inhabitants in the novel are also skeptical of academics and official organizations such as the Monument: “Lebo also knew the only part of town that would be preserved was the Monument, where the eggheads, in return for their cushy income, worked, as Lebo put it, hand in glove with the government, so they couldn’t have cared less that the town would be torn down” ( Topol, 2013, pp. 18–19). That is also why the Terezín inhabitants took action to spread the word about their initiative around the world thanks to Rolf’s reportage: “Rolf 's article was reprinted in many languages, it was published all over the world, so it wasn’t just academics who could speak for Terezín any more, academics installed by a government that didn’t want to pour billions or even millions into a decaying town…” (ibid.). The aim was to seize the chance to speak for Terezín from the perspective of the public. The academics are also portrayed as easily swayed by the wrong incentives. There is tension between the locals who have personal ties to the site and use it for economic gain while fighting for the site and town’s full preservation, and the government alongside the academics, also swayed by economic reasons due to which they wish to reduce Terezín to a monument and educational trails which would cost them less money. Through this conflict, the novel creates an ironic image questioning the general reasons for preservation of WWII sites and their memory and the most appropriate ways of doing so. This, as well as the previous questions related to the limits of Holocaust tourism and commodification is reflected even more in the second part of the novel in Belarus.

Once the revitalization project in Terezín fails due to legal matters, the narrator leaves for Belarus. The squats are destroyed by bulldozers and the inhabitants alongside the visitors are evacuated and taken away by the police. The narrator escapes to Belarus due to his fear of facing charges for participation in the Terezín project, since he is a former convict recently released from prison. He is invited by a Belarusian couple Alex and Maruška to help them with a similar revitalization project in their country. Alex and Maruška are interested in the narrator’s network of contacts who financed the revitalization project in Terezín, in hopes of getting funding for their own project lead by a man known as Kagan. Close to Minsk, the narrator discovers a newly developed museum of horror located in near the site of Khatyn massacre called Devil’s Workshop where he learns about the shocking grotesque and inhumane activities of the Belarusian team working on their exhibition in hopes of bringing European and international dark heritage tourists to Belarus.

In a hyperbolic move by Topol, it is revealed that this fictional museum’s main exhibition is the installation of embalmed bodies of WWII criminals, perpetrators, and witnesses of the awful crimes who have all been wired to an electric mechanism that retell the visitors their darkest memories with the help of their voice recordings playing in the background. The Belarusians wanted to create something shocking that would make their museum recognized outside Belarus, bring crowds of tourists, and “place” the obscured sites of mass graves on the map of Europe. Popular sites of dark tourism such as Auschwitz inspired the Belarusian characters in the novel to aim for a similar outcome and make their local sites of genocide become well-known in the world. The novel thus asks in a satirical manner where the ethical limits of museum experience related to atrocities lie. It also invites its readers to think about the boundaries of turning memory of atrocities into profit. Arthur, a commander of the Belarusian partisan brigade who also works the creation of the Devil’s Workshop museum explains their motivations to the narrator in the following manner:

“Guess who had the most casualties during the war? We did! Guess who had the most people murdered under communism? We did… Thailand: sex. Italy: paintings and seaside. Holland: clogs and cheese. Right? And Belarus? Horror trip, right?...Visit the Devil’s Workshop, the European monument to genocide... Do we have the sea, the mountains, historic buildings? No, all our historic buildings were burned. So we’ll build a Jurassic Park of horror, a museum of totalitarianism. Belarus will get on the map thanks to our bags of bones, our bundles of blood and pus. ( Topol, 2013, pp. 115–116)

Similar to Terezín, the efforts for the Devil’s Workshop project appear to be once again coming from a gain-related perspective. According to the Belarusian characters, the project is supported by the President and by the Ministry of Tourism in Belarus. The purpose of the project is to develop tourism in the country, as its reputation as a tourist destination is very poor at that time. Furthermore, their tourism is also challenged by Russia, as Alex notes: “The Russians are our big brothers. Too big, actually. They want to swallow everything up. Now they’re even muscling in on our tourist industry. It isn’t right” ( Topol, 2013, pp. 131–132). The Belarusian members show a heightened sense of nationalism towards their country which is also one of the motivations behind the Devil’s Workshop. Living in the shadow of Russia motivates them even further to make their country more visible in the world.

Outside the novel, Belarus serves as an extreme example of silenced memory due to the fact that its number of Holocaust victims was the highest among all European countries, but it is hardly remembered in Belarus ( Kovtiak, 2020, p. 53). While the exact number of victims is not known, it is estimated to be between 600,000 to 800,000 (ibid.). A big part of Belarus’s population was Jewish, nearly a third of Minsk’s population, and more than 40 percent of Brest’s ^9^ population (ibid., p. 54). Ghettos were created in both cities, where Jews from Western parts of Europe were transported ( Snyder, 2010, p. 209). In winter 1941–1942, the largest ghetto on the territory of the prewar Soviet Union was located in Minsk, where an estimated 70,000 Jews were held (ibid., p. 228). The ghettos were then liquidated by the Nazis in 1943 (ibid.). Mass shootings and locking up the victims in barns and setting them on fire were the main methods of killing which are also described in Topol’s novel (ibid., p. 242).

While the graveness of the Holocaust in Belarus cannot be denied, the Holocaust was actively silenced in Soviet Belarus. During the Soviet times, the Holocaust narrative was replaced by the Soviet narrative of the Great Patriotic War, powered by different desires including the one “to maintain the myth that Slavs and communists were the primary targets of the Nazis” ( Kovtiak, 2020, p. 52). The possible “inconvenience” of Jewish victim narratives taking over the glorification of Belarusian contribution to the victory over Nazism was one of the reasons why the genocide was not seen as a “separate phenomenon” but rather as “another example of Nazi crimes against civilians” (ibid., p. 52). Another reason was fear of the Holocaust opening up a discussion on the topic of collaboration of Belarusians with the Nazis (ibid., p. 54). In terms of “Stalinist perspective, it was not the killing of Jews that mattered but the possibilities for its political interpretation” ( Snyder, 2010, p. 227). 

In the post-Soviet Belarus, there seems to be a discrepancy and an interesting relationship between what Jan Assmann (2011) distinguishes as “communicative memory” (also described as “vernacular memory” by Bădescu, 2021) which is non-institutional and non-formal and whose transmission is not supported by any official parties, and “cultural memory” which is preserved through various institutions. While the genocide and Holocaust narratives are not necessarily being silenced in Belarusian cultural memory, they are still being suppressed within the official memory culture. There may be public Holocaust monuments but “the narratives that would otherwise give meaning to them are silenced” ( Kovtiak, 2020, p. 52). This type of silencing is present in Belarusian schoolbooks, museums, as well as in its urban landscape ( Kovtiak, 2020, p. 52). The Holocaust memory, however, lives on and emerges in vernacular memory, in hopes of penetrating the official arena and finding their place within it. In her article, Kovtiak explains how after 1991, the Holocaust memorialization has been slowly officially implemented in the country. However, the sites of memory were financed and “created with the help of foreign foundations and private initiatives, not by the Belarusian state” (ibid., p. 54). Even after the dissolution of the Soviet Union in 1991, the Great Patriotic War narrative continues to play an important role for Lukashenko’s rule as he placed it at the core of the state ideology in order to “legitimize his regime” (ibid., p. 54).

Unfortunately, many Holocaust remembrance monuments, sites, and museums in Belarus are hidden away by the landscape, lack of advertisement, or restriction of public access (ibid.). Some WWII mass graves are still unearthed which has also been manifested by the events in 2019 and 2021 when two large mass graves were discovered in Belarus, specifically in the town of Brest and the village of Logoza located approximately 40 km distance from Minsk. ^10^ Regina Simonenko, head of the Holocaust Museum in Brest, has revealed that while the site of this mass grave was known, it was still surprising to see such a huge number of unearthed human remains in the heart of the city ( ABC News, 2019). The mass grave in Logoza was discovered 2 years later, in 2021. It is another mass grave where remains of civilians – senior citizens, as well as women and children – killed by the Nazis during WWII were unearthed ( Jewish News Syndicate, 2021).

Both of these discoveries further bring attention to the importance of Topol’s novel even more than a decade later. These issues are a reflection of Belarus’s difficult heritage which is often hidden away and ignored with the help of the selective process of heritage-making and focus on narratives that emphasize victories and triumphs ( Macdonald, 2009, p. 2). Topol emphasized the magnitude of genocide that took place in Belarus by focusing on one of the many tragedies, the Khatyn massacre, and its shrouded presence, as it can be easily mistaken for or overshadowed by the Katyn Forest massacre in Russia ^11^ because it shares a similar name. Topol’s choice of juxtaposing Holocaust with Khatyn is interesting. The Khatyn massacre can be seen as a Belarusian alternative to a Holocaust site. While Holocaust memory was being silenced in Belarus, this was a narrative that could work within their country in order to create their own tourist attraction. It is mainly because Khatyn and Holocaust narratives both present the Nazis as perpetrators, which aligns with the Soviet narrative of the Great Patriotic War. Furthermore, Khatyn also emphasizes the victimization of Slavic nations due to Nazi crimes. That is why the mentioned “ministry of Tourism” in Belarus supports the museum. The fact that Topol chose the Khatyn massacre alongside the Holocaust allows the readers to not only focus on the local Czech suffering under Nazi regime, but also rethink the Soviet regime and its long lasting influence on Belarus.

While the Khatyn monument in Belarus is a major memorial since the Soviet times, it joins the trend of Soviet myth-making, by abusing the status of victims, turning them into martyrs. Based on the official records, the Khatyn Memorial commemorates victims who “died for the freedom of the USSR”, shifting the focus on the myth of martyrdom ( Lewis, 2015, p. 367). This further demonstrates the inability of Belarusian culture to distance itself from its colonial history (ibid., p. 400). This aspect is also emphasized in the novel through Alex’s statement where he reflects on the relationship Belarus has with Russia, which I have cited above. Lewis claims that literature can be powerful to present situations that are at odds with the “state’s triumphalism”, and that “Topol’s transnational treatment of wartime victimhood may contain a key to enabling Belarus to overcome the lingering legacy of Soviet-era martyrdom” (ibid., p. 367).

In Topol’s novel, the Belarusian characters seek financial support for their memorialization project from abroad, specifically from the Czech Republic, while trying to integrate the communicative memory into the cultural one. In the real world, there have been several projects in Belarus created by nongovernmental organizations that recreate history using vernacular sources, such as photos or interviews with witnesses and survivors, which were ignored in the official narratives (ibid., p. 53). In the novel, the creators of the Devil’s Workshop were inspired by the work done by Lebo’s team in Terezín, which lead them to believe that oral history and the story itself is what matters the most. Thus, they also decided to rely on vernacular sources in their own twisted way, by basing the exhibition on authentic testimonies told by the voices of mummified victims and perpetrators. This aspect of the novel further showcases its dark, satirical nature and questions the boundaries of ethics in relation to cultural memory, memory entrepreneurship and dark tourism.

Furthermore, by looking closer at the politics of memory in both the Czech Republic and Belarus, it can be said that Topol’s novel is a satirical reflection of both countries’ way of dealing with their own difficult heritage. In comparison, both Czech and Belarusian Holocaust memory have been avoiding the element of Jewish victimization that lies at its core. While the former emphasizes their own victimhood, the latter focuses on myths of victories and triumphs and martyrdom. As they share both similarities as well as differences in the way external pressures, both Eastern and Western, have shaped their stance towards the Holocaust, they offer a fruitful ground for juxtaposition in Topol’s novel. By juxtaposing the Czech Republic with Belarus, Topol turns his novel into a transnational text that can allow readers to reflect on the past, memory cultures, as well as politics of memory of different countries in comparison.

## *The Devil’s Workshop* and the myth of the East

Juxtapositions are one of the key elements on which *The Devil’s Workshop* is built. Topol juxtaposes Eastern and Western Europe based on a purposefully hyperbolic division of Europe in terms of aspects such as memorialization cultures, dark tourism, and the way of dealing with difficult heritage. The division of Europe is created through the characters’ different, non-aligning points of view in relation to genocide memorialization efforts and practices, coming to terms with the past, and the personal feelings of “superiority” and “inferiority”.

Three main points of view can be observed. The first point of view is represented by a Swedish girl Sara. Based on her perspective on the Terezín revitalization project, she highlights Sweden as the more superior Western European country in comparison to the Czech Republic. She also expresses her aversion towards communism and its lasting influence on the countries that were under the regime. She describes its influence as “filthy”: “Culturally we’re totally different! I mean, I’m completely untouched by communism, but you’re up to your neck in filth!” ( Topol, 2013, p. 40). When talking to the narrator, Sara asks whether he can guess why she likes it in Eastern Europe and why she feels so good there. After his incorrect guess, Sara explains that she likes it in the East because she feels “superior” there and while in the East everybody seems to have complexes because of who they are and where they come from, she only has her personal complexes that she needs to deal with ( Topol, 2013, p. 44). In the novel, Sara and other visitors of Terezín are described as “drops of water, seeped into the underground currents of the mysterious East” ( Topol, 2013, p. 32). There are several words that qualify the West and East in the novel, including expressions such as the “superior West” and the “mysterious East”, which are reminiscent of Edward Said's theory of orientalism, further emphasizing the cultural differences of the aforementioned European countries in relation to their geographical position as well as their ties to former political regimes ( Said, 2003, p. 26).

The second point of view is looking from within the East towards the West represented by the Belarusians’ way of seeing parts of Europe that lie in the western direction including Poland, Germany, and Czech Republic. They view these countries as superior in the way they more openly deal with their difficult heritage and turn it into sites of dark tourism. In the novel, Auschwitz is highlighted by the Belarusians as an ideal example of transforming a genocide site into a popular dark tourism site where the dark past is revealed to thousands of tourists every year. ^12^ By referring to Auschwitz as an ideal and juxtaposing it with Belarusian memory efforts and narratives, Topol places emphasis on the situation of genocide memory in Belarus and attempts to show how it tries to break free from the “historical amnesia” ( Etkind, 2013, p. 10) and “enforced forgetting” ( Lewis, 2013, p. 196) – two prevalent approaches toward memory in Belarus – setting in motion the endeavor for memorialization and remembrance.

The third point of view is represented by the narrator. Based on all the dialogues taking place throughout the novel, he and his homeland – the Czech Republic – assume a central position in this threefold juxtaposition, as it is the place where the East and the West meet. While the narrator sees the Eastern Europe based on the example of Belarus as more wild and problematic than his own country due to its political situation and the dubious characters he meets, he also gets to experience the feeling of inferiority from a western country representative – Sara from Sweden, who points out that the way his homeland is approaching its difficult heritage is wrong and shameful. Due to the superiority and inferiority dialogues the narrator has with the Belarusians and Sara, he and his home country as a result assume the position of Central Europe.

Thus, several scales are present. What is considered as Eastern from the perspective of Sara, differs from that which is considered Eastern by the narrator, Belarusians, and many others. The Belarusians are looking up to what they understand as “Western” and more developed Europe represented by Poland and the Czech Republic. On the contrary, Sara looks down upon the Czechs and their lack of interest towards the Holocaust and its memorialization. Through her, Sweden represents a superior kind of Western Europe as an ideal. The novel makes the “West” become a metaphor that has little to do with geographical location. It becomes a metaphor for a “better, more developed” place unplagued by difficult heritage. Moreover, the East becomes a metaphor for something “torpid”, “uninterested”, and “inferior” that is burdened by its difficult heritage from whose clutches it is trying to escape. The will to escape the East to the extent that it becomes a myth is illustrated through an anecdote told by Sara. She explains how she attempted to find and visit the East. She began her search in Slovakia, kept on asking and going eastwards until she arrived in Vladivostok where she got out of the train and was told that she has just arrived in the “honest-to-God end of the West”, at “the end of Europe” ( Topol, 2013, pp. 42–43). Through a clever, satirical anecdote, Topol manifests the undeniably stigmatized view of being associated with the East, which is also underlined by the subtle reference to Said’s orientalism.

All the topics arising within the novel as well as its border-crossing juxtapositions create a transnational web of different stances towards politics of memory and point to how Europe is still divided despite the ongoing Europeanization efforts. Thanks to the narrative’s transnational quality and several interwoven concepts that transcend the borders between Eastern, Central and Western Europe, the novel becomes more open to readers from all over the world. However, in order to confirm how the international audience received the novel, its reception must be taken into account.

## The author, translator, and reader

In his statement used as an epigram for this article, Topol makes a clear distinction between a novelist and journalist and their relationship with their readers. He as an author believes in writing without regard of his readers’ origin or age or the need to cater to the popular trends. In the same interview he states further:

Whether it’s interesting for “the young” is of an equal importance to me as whether it’s interesting for “the British”. My books are published in Croatia and Poland and nobody asks if it is interesting for the Croatians or for the Poles. I understand. English dominates the world. I am not rushing anywhere… I am satisfied with writing for the old Poles and Croatians till the end of my life… I have to make fun of that all. The last person I think of when writing a book is the reader. I would, clearly, feel sorry if a few of my friends found the book completely stupid, but I can’t write for a particular person, I would feel as if I were pandering myself. ( Topol, 2010a)

Topol’s novels traditionally make the readers experience events through his local, Czech, perspective which is reflected in his writing style, selected themes, and the use of the typical Czech dark humor. The question of whether a foreign, or transnational readership would be able to comprehend, or enjoy his work is secondary to him. Despite Topol’s views on the foreign reader, I want to argue that with his novel *The Devil’s Workshop* he is going against the grain of his view and writing it not for the local, but transnational audience.

As my analysis has shown, thematically, *The Devil’s Workshop* has a great potential to speak to transnational audiences, even if there may be some stumbling blocks linked to Topol’s writing style. Thus, it is interesting to explore the reception of the novel to see how different the readers around the world respond to and interpret it. The aim of the study of the reception is to explore how the novel, which is arguably written from a local perspective for a local audience, can have a place in a transnational arena, despite its peculiar aesthetics.

Topol’s writing style bears many distinct characteristics which make his work difficult to translate and understandable to foreign readers. He usually writes in colloquial language, makes subtle references to places and events of historical importance, and intertwines fact and fiction in a manner which makes it hard to distinguish where the former begins and the latter ends ( Chew, 2015, p. 11). His writing also “pushes the limits of the Czech language” to the extent that the Czech publisher of his novel *Sestra* ( Topol, 1994) had to include a note stating that the liberties the author had taken with language were part of his poetics, not a product of poor grammar ( Woods, 2016, p. 83). In 2000, *Sestra* was translated into English under the name *City, Sister, Silver* by Alex Zucker – a translator who brought several Topol’s books to an English-speaking audience – who managed to transpose Topol’s poetics to the English language while maintaining its effects (ibid.). However, some reviewers have suggested that even though Zucker’s rendering of the novel was successful, it would still be difficult for the non-Czech readers to understand and orientate within the cultural context due to the “mixture of the foreignness of context and the foreignness of the language” (ibid., p. 84).

Another common trait of Topol’s writing, including *The Devil’s Workshop*, is the use of different means to make his text evoke the feelings of uncertainty and unreliability, such as withholding information, providing contradictory information, or choosing a passive narrator – which as a result significantly expand the readers’ possibilities of interpretation and misinterpretation ( Dědinová, 2014, p. 110). Adding these aspects on top of Topol’s local perspective in *The Devil’s Workshop* may pose as a barrier for transnational audiences of the novel. The extent to which readers read the novel differently remains to be seen.

To aid the reader, the translator of *The Devil’s Workshop*, Zucker, has supplemented the novel with a “Translator’s Afterword” in which he provides some information about the setting of the first part of the novel set in Terezín and explains the important aspects of Belarusian past events that are referenced in the novel. The afterword also contains Zucker’s own reflection on Topol’s way of blurring the line between fact and fiction and helps the readers to navigate throughout the text by unearthing the factual sources behind some of the anecdotes and passages in the text. Another helpful paratext present in the book is the “Author’s Acknowledgements” which, despite being fairly short, contains some references to texts and victims’ testimonies which Topol had drawn information from when writing the novel. 

Fact and fiction aside, the most striking aspect of Topol’s aesthetics in *The Devil’s Workshop* is dark humor and satire which can also pose some challenge when translating and understanding his work outside the local arena. Topol and Zucker explained that dark humor is a kind of “trademark” of Czech literary tradition, which can complicate the way the foreign readers understand or “relate to” the text ( Topol & Zucker, 2023). Inclusion of humor in the narratives about the Holocaust can be disconcerting as some readers may find it inappropriate or ill-suited, however, humor has been a functioning feature of Holocaust literature ( Cory, 2004, p. 197). ^13^ The employment of humor within dark history narratives can be explained by various reasons. Humor can be used to “mark the boundaries between different orders of reality”, establish “nuances of credibility in incredible circumstances” or to function “as resistance” or “protest” (ibid., p. 195; Kaplan, 2002, pp. 343–356). Sometimes, it can be the “authors’ conviction that laughter is the best response to historical trauma” as it allows people to “recognize the absurdity of such historical events as wars and the smallness of an individual against the circumstances of history… but also show a desire to overcome the pain and disappointment which history brings and master our past” ( Mazierska, 2011, p. 20). Mazierska also explains that “comic elements have an important potential to evoke a more active response from the audience than the more usual genres like classical tragedy, realistic drama, or documentary” (ibid., p. 209).

One of the most typical examples of humor present in Czech film and literature is “Kafkaesque black humor” ^14^ which can be also recognized within *The Devil’s Workshop* (ibid., p. 210). Topol’s writing in this novel is closely tied to Kafka in style as well as the way humor works in Kafka’s work ( Woods, 2016, p. 86). Furthermore, Woods notes that Kafka is generally regarded as difficult to grasp, translate, and “master” and this is another common point of reference between him and Topol (ibid.). Despite the fact that Zucker has been closely working with Topol for many years and mastering the art of interpreting Topol’s work for English language audiences, the dark humor and grotesque as well as the local settings of the story are still difficult for the readers to understand.

## *The Devil’s Workshop* and the local readers

Based on the reviews it seems that after reading *The Devil’s Workshop* Czech readers were able to reflect on memory cultures in their own as well as in other countries. While they were familiar with different sites of genocide such as Terezín in the Czech Republic and Auschwitz in Poland, they had never heard much about the large scale of the WWII related atrocities that took place in Belarus: “We have Terezín as one of our still living historical horrors. Here we suddenly learn that elsewhere these horrors were many times more massive and horrible… and we do not have them in our consciousness, or we do not even know about them” (Databazeknih.cz Reviewer 1, 2017). It is clear that the Czech readers gained insights into Belarusian history and memory and viewed the novel as a source of important information, despite its fictional plot: “Shockingly, the fact that Belarus had the highest number of deaths during the Second World War is not much talked about. It is also shocking that mass graves are still being found there. I am glad that this information got to me through the book. Better late than never” (Databazeknih.cz Reviewer 2, 2023). The fact that this and other reviewers became aware of newly unearthed WWII mass graves which they mention in their comments, suggests that the novel also inspired them to look outside its fictional world and seek parallels with the world outside the book. Another reviewer explains that according to their view the novel “is not meant to be a grand, reader-absorbing epic narrative so much as a semi-documentary, a text brimming with facts and statistics, leaving behind a reader enlightened and pondering the content conveyed” ( Říha, 2009). This shows that Topol’s way of intertwining fact and fiction appears to be an effective way of creating more interest in history and cultural memory.

Czech readers also discussed their views on the role of the narrator, who does not show much interest in the past and in memory. According to one reviewer, reading the novel from the disinterested narrator’s point of view results in “an impressive contrast between the non-biased description and the insane horror of what he describes” (Databazeknih.cz Reviewer 3, 2017). As the main character does not present any particular worldview and does not understand the deeper political issues surrounding him due to lack of knowledge, the readers also see his role as a vessel for their own self-reflection. Another reviewer explains this in more detail:

Retrospective views into his [the narrator’s] tragically marked life give the reader a unique opportunity to perceive the realities of the time without being distracted by excessive participation, which would probably be unavoidable in a more psychologically refined character” ( Horváth, 2017).

Thus, Topol’s choice of an unrefined narrator who is learning about the world and state of memory in different countries over the course of the novel, and who does not have any strong opinions on most matters, was generally seen as a positive feature, giving the readers more space for self-reflection on the issues portrayed in the novel.

 Most of the time, the readers’ discussion of the novel revolves around its references to reality. The readers mostly interpreted it as a reflection of real-life events and the state of the politics of memory. One reviewer, for example, interpreted the novel within the context of competitive memory between Eastern and Western Europe: “…the message of this short novella is huge, completely transcending the work itself; the message of ignorance of eastern disasters… at the expense of… “more tempting” western tragedies. At the same time, it opens up the contemporary problems of lack of freedom in Belarus and, by extension, in Russia...” (Databazeknih Reviewer 4, 2022). The novel’s transnational quality opens up the perspective of the local readers and provokes them to look at the fragmentation of Europe in terms of WWII memory and invites them to be perceptive of what Mälksoo describes as the “noticeable bias in favour of Western” European history and memory to the detriment of Eastern European in writing Europe’s history ( Mälksoo, 2009, p. 672).

There were many questions that the novel raises for its local readers such as: “… is it possible to compare the size of the horrors? How is Terezín a lesser evil than the repeated horrors in Belarus? And how much can one profit from this historical evil? To what extent does the commercialization of that evil become evil itself?” (Databazeknih.cz Reviewer 5, 2022). The reviews raise the issue of what Michael Rothberg calls “competitive memory” (2009, p. 9), which is in the novel reflected in the way the Belarusian characters are burdened by the ignorance of the outside world, as the memory of the atrocities that took place on their land is overshadowed by other, more infamous ones. However, through the novel’s reception, the competitive memory portrayed in the novel is actively turned into “multidirectional memory” ( Rothberg, 2009). Rothberg describes the model of multidirectional memory as one that allows to produce more memory within the public sphere through contrast and interferences of memories of different events (2009, p. 202). In this case, the reception of the novel where various atrocities are compared outside of the fictional world of the novel, proves that the comparison and potential competitiveness of these memories helps to actively produce greater awareness and debates on the matters that would otherwise stay in the shadows. By bringing the attention of Czech as well as foreign readers to the Khatyn Massacre and comparing it with other sites burdened by difficult heritage, the novel eventually produces more avenues for creating memory in its readers and in their reflection on the issues portrayed.

In terms of the aesthetics, Czech readers noticed some important differences in poetics and writing style between the first and second half of the novel. The first part of the novel that focused on Terezín felt more complicated and included more metaphors and figurative language; some passages were difficult to follow even for the local readers: “The first part taking place in Terezín is more imbued with metaphors and poetic rhetoric, it is full of digressions into one’s own imagination, to the point of getting lost in it” (Databazeknih.cz Reviewer 2, 2023). The second part of the novel was perceived as simpler and more direct in its language style and the way it presented information: “The second part, set in Belarus, is written in a straightforward manner. Here, the author does not leave gaps open to the reader’s interpretation, but serves up unpleasant raw reality, sometimes lightened by morbid humor” (ibid.). These differences can be seen as the manifestation of the fact that Topol’s text is written from a Czech local perspective with Czech readers in mind. In the first part, he allows himself more artistic freedom, providing the readers with a certain degree of challenge related to the references it makes. However, when the plot of the novel takes the protagonist to Belarus, Topol offers them a little more guidance. The readers can feel that they are no longer treading on local territory, as the writing style of the novel changes, adapting to the transnational lens and embedding more information for the Czech readers to grasp the plot. When reading the part of the novel set in Belarus, the readers feel as if they were taking a tour to Belarus with Topol as their guide. In this scenario, the readers look at Belarus as the epitome of “the East” from their assumed “Central European” point of view.

The Czech reviews also show how reading the novel in its original language can influence its interpretation. One reviewer was fascinated by Topol’s anecdote about the non-location of Eastern Europe. They explained that it is hopeless to look for Eastern Europe from within itself, as nobody wishes to identify with it. Rather, Eastern Europe could be every place which has not yet properly dealt with its past. By paying attention to double meaning of the Czech word for “East” – *východ* – which means both “East” as well as “exit” ( Říha, 2009), this reviewer read the novel as a metaphor for Topol’s obsession with looking for Eastern Europe as well as a way out of it. 

## International reception of *The Devil’s Workshop*

The international audience ^15^ was mostly appreciative of the novel with some slightly critical remarks about the style of the prose that some found “too ordinary” to the extent that it left them dissatisfied ( Malone, 2013). Many reviewers found the novel’s structure and its setting in two different national contexts fascinating, allowing them to reflect on the past and present of these countries and compare their dark heritage sites. They felt that the juxtaposition made lesser-known narratives that deserve attention more visible. The international readers also touched upon the question of competitive memory as the novel raised their awareness of the inequalities of public remembering and limited public knowledge about the dark past of some European countries in comparison to others. Several readers reflected on their pre-existing knowledge of the historical events evoked in the novel and stated that they were quite unaware of Belarusian history and the Khatyn massacre. Many reviews found that the novel was revealing both in terms of the past histories as well as issues of political memory.

A strange tale that engages with how we try to come to terms with the heritage of a systematic cruelty and evil like the Holocaust, as well as how it can become politicised and even commercialised. It also opened my eyes to the lesser known racial mass murders carried out in Belarus under the Nazis. (Goodreads Reviewer 1, 2023)

The juxtaposition of sensation-seeking tourists with those who are truly interested in the past or search for healing in the novel was noticed by some of the readers and made them reflect on these different approaches to dark heritage. For example, one reviewer mentions a social media post that came to their mind when reading the novel: “Recently, a screencap from a Swedish high schooler’s Facebook made the rounds on social media. It pictured her and her friends on a school trip to Auschwitz, dancing under the ARBEIT MACHT FREI sign, captioned with “Refuse to be PC, lol!”” (Goodreads Reviewer 2, 2015). The reviewer juxtaposes these careless tourists with *The Devil Workshop*’s character Sara, reflecting on the significance of Sara’s attempt to turn Terezín into a site of remembrance while expressing disapproval of disrespectful Auschwitz visitors. This is one of the examples of how the novel prompts the readers to compare the happenings within the novel with those that surround them in the real world. Overall, the reviews demonstrate that Topol’s focus on juxtapositions that portray and contrast different approaches to dark heritage, practices of memorialization, genocide tourism and memory competition on a transnational scale is a fruitful method for foregrounding the past and allowing people to learn more about it. On that matter both local as well as international reception agree.

 While *The Devil’s Workshop* proves to have a positive influence in raising awareness of the dark chapter of the European past, the readers also signaled their problems in understanding the novel. According to some, the author did not provide enough information about the historical context that would be useful for the readers for grasping the story. Several reviewers felt that a lot of important context content was left out by Topol because the novel was originally written for Czech audiences who have more knowledge of their country’s history and the site of Terezín. The lack of historical context in the first part of the novel proved to be an issue for non-Czech readers: “The first part, with the introduction of the characters and the somewhat impenetrable portrait of Terezín then and now, was partly incomprehensible to me… and many things made sense only after reading the second part” (Goodreads Reviewer 3, 2015). Accordingly, the reviewers really appreciated two paratexts by the translator Zucker and Topol himself, discussed above, that filled in several “gaps” in their historical knowledge, allowed them to make sense of the events and resolved some of their confusion ( Vane, 2015). Thus, as the narrator himself crosses borders between countries, Topol allows the readers to cross the border between his usual, more impenetrable, style of writing manifested by the inattentive stance towards his readers, and a more guided and transnationally approachable style of writing.

 The reviewers also discussed the novel’s aesthetics and language and how they influenced their reading experience. They felt that Topol’s writing style and use of language can destabilize the reader. According to the reviewers, the incoherent narrative, the way that many events were stripped of context and left without any explanation, the “short and sharp sentences”, as well as the use of direct speech without the proper punctuation evoked feelings of disorientation, instability, uncertainty, and discomfort ( Messenger, 2014). As the novel presents the readers with a museum of past atrocities and totalitarianism, its aesthetics further emphasize the horror element. Topol’s use of aesthetics can affect the readers and make the reading emotionally challenging, similar to the Holocaust museums that attempt to create an emotionally challenging experience through different strategies of exhibition design ( Sodaro, 2018). This way, the readers’ visit of the Devil’s Workshop Museum within the narrative becomes intensified through Topol’s writing style, as it is easy to get lost and disoriented amidst a place of horror.

 Many international readers discussed the presence of dark humor and satire within the novel. While, as I have shown, Topol has argued that these aspects could have been challenging to translate and difficult to understand for transnational readers, the opposite seems to be the case based on the reviews. Many reviews mentioned Topol’s satire and dark humor in a positive way: “ *The Devil’s Workshop* made some good points via a very readable satire / adventure that was far from dreary” (Goodreads Reviewer 4, 2014). They commended the author for having the courage to approach dark history with humor and for his satirical portrayal of the “Disneyfication” of Holocaust: “It takes a bold writer to approach two examples of man’s inhumanity to man with dark humour, but the author of this short book achieves this allowing the reader to feel uncomfortable as he confronts the greatest horrors of the twentieth century” (Goodreads Reviewer 5, 2017). The role of satire in a dark story with ties to reality was a welcome tool that allowed the readers to navigate the pool of different conceptual ideas rooted within the novel. Satire and the feelings of discomfort that the novel evokes can together create a space for readers’ self-reflection: “it definitely brought up some interesting issues, particularly in its satirization of dark tourism and how we, as a community, remember the Holocaust” (Goodreads Reviewer 6, 2013). This conclusion is also apparent from other reviews such as the following: “The Devil's Workshop is a very dark satire about the Holocaust business, that is, the business of how we collectively choose to approach our worst behaviors as a species” (Goodreads Reviewer 7, 2016).

Finally, some reviews also discussed Zucker’s translation of the novel. A reviewer pointed out that while it is difficult to judge a translation without having read the original, the translation was in their opinion successful: the text read naturally and the main points that were expressed through satire were easy to comprehend (Goodreads Reviewer 4, 2014). The positive stance of the reviewers towards the translation of the novel and towards the use of satire seemingly confirms the idea that satire and dark humor can be a successful response to discussing historical trauma and difficult heritage that works on a transnational level.

## Conclusion

Topol’s novel *The Devil’s Workshop* brings readers’ attention to many important issues and discussions related to the memorialization efforts and practices in the Czech Republic and Belarus mainly through juxtaposition and satire. Topol’s novel offers a satirical reflection of both countries’ ways and preferred narratives of dealing with their own difficult heritages. Since the Czech Republic and Belarus share similarities and differences in the way external pressures, both Eastern and Western, have shaped their stance towards the Holocaust and atrocities committed by the Nazis, they offer a solid ground for juxtaposition in Topol’s novel. Through it, Topol turns his novel into a transnational text that can allow readers to reflect on the past, memory cultures, as well as the politics of memory of different countries.

Even though Topol writes novels from a local Czech perspective with a strong sense of traditional dark humor without any regards to how his work could be translated or understood by foreign readers, the transnational context of this novel challenges this Topol’s common practice and allows the transnational audience to gain new insights and to appreciate his work.

The analysis of the novel’s reception has revealed that it was received positively, not only by the local, but also transnational readers, despite some minor issues. The reception was more positive thanks to the inclusion of paratexts in form of the “Translator’s Afterword” and “Author’s Acknowledgements” which provided more information in terms of missing historical context and shed greater light on the authenticity of the events mentioned in the narrative. Many of the novel’s aesthetics and aspects, such as the use of satire, or the employment of an uneducated narrator allowed for further self-reflection for the readers in relation to portrayed issues. The role of satire and dark humor served as a compass, guiding the readers through the multitude of conceptual ideas presented within the novel while expanding the available room for readers’ reflection.

 Both local and international reviews have offered many insights into how the novel can be interpreted, and how it can be read metaphorically. Based on events in recent years, it is clear that the novel is still worth reading as it can raise awareness and point towards timely issues. These include the ways Eastern European countries have been dealing with their difficult heritage, the ways how past can be easily silenced and hidden away, the presence of yet-to-be-uncovered mass graves, or the effects of memory entrepreneurship. Thanks to these aspects, as well as Zucker’s successful rendition of the novel, *The Devil’s Workshop* can be considered as a significant contribution to the transnational arena of literature.

## Ethics and consent

Ethical approval and consent were not required.

## Footnotes

^1^This novel was first published in the Czech language in 2009 under the title *Chladnou zemí*. Throughout the article, I will mostly refer to the English translation published in 2013, except when discussing its local reception.

^2^Not to be confused with Katyn forest massacre in Poland. The second part of the novel refers to Khatyn massacre which took place on 22 March 1943. During it the 118th Schutzmannschaft auxiliary police battalion killed Belarusian villagers in Khatyn, 50km to north-east from Minsk. In the translator’s afterword to the English translation of the novel, Zucker explains that Topol faithfully depicts the killing methods that were used in Khatyn: the soldiers herded people into barns and set them on fire while shooting everyone who tried to escape ( Topol, 2013, p. 164).

^3^Within this article, I define local readers as mostly Czech readers with inclusion of Slovak readers due to the close historical, lingual, and cultural proximity of the two nations.

^4^Topol's first novel *Sestra* was published in 1994 ( *City, Sister, Silver, 2000*). It is a dynamic adventurous love story that takes place after the Velvet Revolution and focuses on the ''opening'' of Europe and life in the former Czechoslovakia in the late 1980s and early 1990s'. Another important Topol's novel published in 2005 called *Kloktat Dehet* ( *Gargling with Tar*, 2010b) follows a young orphan who is trying to survive in Czechoslovakia during 1968 when the Soviet troops invaded the country.

^5^According to Anja Tippner (2021), 21 ^st^ century works of Czech authors addressing WWII topics such as the Holocaust or the expulsions of Germans from Czechoslovakia, have shifted from their previous tendency to treat the past from a personal perspective to considering these topics as “entangled and transnational phenomena” (pp. 80–81). Tippner also uses Rebecca Walkowitz’s concept (2015), to mark *The Devil’s Workshop* and other Czech literary works as ‘born translated’, due to their transnational characteristics which make them more appropriate for entering the international arena than ever before.

^6^I have created my own database of these reviews and manually coded the gathered qualitative data in order to highlight the most prevalent stances of both local and transnational readers. In total, a number of 100 reviews were examined in order to answer the posed questions. I decided not to disclose the usernames of the reviewers taken from the aforementioned platforms. Instead, I have assigned them pseudonyms. I only use names/usernames of those reviewers who published their reviews on their own blogs or websites. Reviews written in Czech and other languages written by international readers were translated into English by me.

^7^Terezín (Theresienstadt) is a fortress town in the North-Western part of the Czech Republic built in 1784 on the orders of Emperor Joseph II for defense against possible Prussian invasion ( Lennon & Smith, 2004, p. 17). After the occupation of the Czech territory in 1940 by Nazi Germany, Terezín’s Small Fortress was turned into a Prague Gestapo prison and the town became a Jewish ghetto (Ibid.). Terezín served many purposes during WWII, being “a prison, transit camp, death site and a propagandistic camp” for about 140 000 prisoners, men, women, and children from different European countries (Ibid.). The prisoners were brought in before extermination camps further east were ready to take them in for liquidation (Terezín Memorial’s official website). Most of them were taken to Auschwitz-Birkenau (ibid.). Out of the estimated 87 000 individuals who were brought to Terezin, only around 3800 were lucky to live until their liberation (ibid.). According to the information listed on Terezín Memorial’s website, the estimated number of prisoners who died there due to hunger and harsh conditions is about 35 000. The Terezín concentration camp functioned from 24 November 1941 to 9 May 1945 until the arrival of the Soviet forces

^8^On June 10 in 1942, the inhabitants of the Czech village called Lidice were murdered by the Nazis and the village was razed to the ground. The massacre was carried out as a revenge because the village was suspected of collaborating on Operation Anthropoid, during which assassination of a high-ranking SS officer, Reinhard Heydrich, was carried out the same year. ( Sniegon, 2014, pp. 203–204)

^9^Brest (formerly Brest-Litovsk) is a Belarusian city located on its border with Poland.

^10^The mass grave of more than 1,200 victims in Brest was found in February 2019 by construction workers who were going to build a luxury apartment complex there. Many of the victims' bodies were those of women, teenagers and babies who had bullets in their skulls ( ABC News, 2019).

^11^This atrocious event took place in 1940 during which thousands of Polish military officers were brought by the Soviet secret police to the Katyn Forest, where they were killed and buried.

^12^Auschwitz has been widely recognized as “the most familiar killing site” of what Timothy Snyder calls “bloodlands” and it has become a well-known Holocaust symbol ( Snyder, 2010, p. VIII).

^13^The representations of the Holocaust that implemented “satire, irony, self-deprecating humor, and grotesque and gallows humor” were present in some Czech novels and films already in the 1940s ( Holý, 2017, p. 208).

^14^Kafkaesque humor can be described as the humor of the absurd. It is a complex and critical type of humor, usually tied to political thinking and ideas of freedom. ( Vardoulakis, 2016)

^15^The reviews used for my analysis are from readers who have very different backgrounds. There were avid readers and historians among them, as well as casual readers with interest in the topic of WWII and the Holocaust. It was clear that their background greatly influenced their interpretation of the novel. Information regarding their national background was not always available, but those identified were mostly from different parts of Europe and the United States.

## References

[ref-3] Assmann J : Communicative and cultural memory.In: *Cultural Memories.*edited by P. Meusburger, M. Heffernan, and E. Wunder. Dordrecht: Springer,2011;4:15–27. 10.1007/978-90-481-8945-8_2

[ref-4] Autry R : Desegregating the past. New York Chichester, West Sussex: Columbia University Press,2017. 10.7312/columbia/9780231177580.003.0001

[ref-5] Bădescu G : Homelands and dictators: migration, memory, and belonging between Southeastern Europe and Chile. *J Contemp Eur Stud.* 2021. 10.1080/14782804.2021.2014305

[ref-6] Beech J : Chapter 11. ‘Genocide Tourism’. *The Darker Side of Travel: The Theory and Practice of Dark Tourism.* edited by Richard Sharpley and Philip R. Stone, Bristol, Blue Ridge Summit: Channel View Publications,2009;207–223. 10.21832/9781845411169-012

[ref-7] Chew G : 'Dissidence' in holocaust memorials in literature: Jáchym Topol’s *The Devil’s Workshop* ( *Chladnou zemí*). *Cent Eur.* 2015;13(1–2):87–102. 10.1080/14790963.2015.1107325

[ref-8] Cory M : Comedic distance in holocaust literature. *Literature of the Holocaust.* edited by Harold Bloom, Chelsea House Publishers,2004;193–204. Reference Source

[ref-9] Dĕdinová T : Prostředky vyjádření nejistoty v prozaickém díle Jáchyma Topola. *Bohemica litteraria.* 2014;17(2). Reference Source

[ref-10] Etkind A : Warped mourning: stories of the undead in the land of the unburied.Stanford, CA: Stanford University Press,2013. Reference Source

[ref-12] Hirsch M : The generation of postmemory: writing and visual culture after the holocaust.Columbia University Press,2012. Reference Source

[ref-13] Holý J : Nontraditional images of the holocaust in Czech literature and cinema: comedy and laughter. *Holocaust Studies.* 2017;23(1–2):208–221. 10.1080/17504902.2016.1209835

[ref-14] Horváth D : Výlet do skanzenu masových popráv v sprievode Jáchyma Topola. *Medziknihami.sk.* 2017; Accessed 16 March 2024. Reference Source

[ref-15] Hucal S : When a selfie goes too far: how holocaust memorial sites around Europe combat social media disrespect. *ABC News.* 2019; Accessed 20 November 2023. Reference Source

[ref-16] Kalousová E : You survived? We were told all jews were gassed’: Jews, Czechs, and the memory of the Holocaust. *The Journal of Holocaust Research.* 2022;36(2–3):186–200. 10.1080/25785648.2022.2071195

[ref-17] Kaplan L : ‘It will get a terrific laugh’: on the problematic pleasures and politics of Holocaust humor. *Hop on Pop.* edited by Henry Jenkins *et al.*, Duke University Press,2002;343–356. 10.1215/9780822383505-022

[ref-18] Kousha K Thelwall M Abdoli M : Goodreads reviews to assess the wider impacts of books. *J Assoc Inf Sci Technol.* 2017;68(8):2004–2016. 10.1002/asi.23805

[ref-19] Kovtiak E : Bringing back the silenced memories, (un)official commemorations of the Holocaust in Belarus. *Baltic Worlds.* 2020;13(4):50–60. Reference Source

[ref-20] Lennon JJ Smith H : A tale of two camps: contrasting approaches to interpretation and commemoration in the sites at terezín and Lety, Czech Republic. *Tourism Recreation Research.* 2004;29(1):15–25. 10.1080/02508281.2004.11081427

[ref-21] Lewis S : Toward cosmopolitan mourning: belarusian literature between history and politics. *Memory and Theory in Eastern Europe.* edited by Julie Fedor. Palgrave Macmillan US,2013. 10.1057/9781137322067_10

[ref-22] Lewis S : Overcoming hegemonic martyrdom: the afterlife of khatyn in belarusian memory. *Journal of Soviet and Post-Soviet Politics and Society.* 2015;1(2):367–401. Reference Source

[ref-23] Lichtenstein T : 'It Is not my fault that you are Jewish!': Jews, Czechs, and the memory of the Holocaust in film 1949–2009. *Dapim: Studies on the Holocaust.* 2016;30(2):117–141. 10.1080/23256249.2016.1166591

[ref-1] Mass grave of holocaust victims uncovered in Logoza, Belarus. *Jewish News Syndicate.* 2021; Accessed 3 November, 2023. Reference Source

[ref-24] Macdonald S : Difficult heritage: negotiating the nazi past in nuremberg and beyond.New York: Routledge,2009. Reference Source

[ref-25] Mälksoo M : The memory politics of becoming European: the east European subalterns and the collective memory of Europe. *Eur J Int Relat.* 2009;15(4):653–680. 10.1177/1354066109345049

[ref-26] Malone T : Review of the devil's workshop, by jáchým topol (Review). *Tony's Reading List.* 2013; Accessed 16 March 2024. Reference Source

[ref-27] Mazierska E : European cinema and intertextuality: history, memory and politics. Palgrave Macmillan,2011. 10.1057/9780230319547

[ref-28] Messenger T : Review of The Devil's Workshop, by Jáchým Topol.2014; Accessed 16 March 2024. Reference Source

[ref-2] More than 1,000 bodies discovered in Belarus mass grave a dark reminder of holocaust. The discovery was made near the polish border. *ABC News.* 2019; Accessed 3 November, 2023. Reference Source

[ref-29] Němcová Banerjee M : Czech laughter between hašek and kafka. *World Literature Today.* 1985;59(1):14–17. 10.2307/40140524

[ref-30] Říha I : Topol, Jáchym chladnou zemí 2. *iLiteratura.cz.* 2009; Accessed 16 March 2024. Reference Source

[ref-32] Rothberg M : Multidirectional memory: remembering the holocaust in the age of decolonization.Stanford: Stanford University Press,2009. 10.1515/9780804783330

[ref-33] Said EW : Orientalism.London: Penguin Books.2003. Reference Source

[ref-34] Sniegon T : Vanished history: the Holocaust in Czech and Slovak historical culture.Berghahn Books. JSTOR,2014;18. 10.3167/9781782382942

[ref-35] Snyder T : Bloodlands: Europe between hitler and stalin.New York: Basic Books,2010. Reference Source

[ref-36] Sodaro A : Exhibiting atrocity: memorial museums and the politics of past violence.Rutgers University Press,2018. 10.2307/j.ctt1v2xskk

[ref-38] Tippner A : Postcatastrophic entanglement? Contemporary Czech writers remember the holocaust and post-war ethnic cleansing. *Mem Stud.* 2021;14(1):80–94. 10.1177/1750698020976463

[ref-70] Topol J : Sestra.Brno: Atlantis,1994. Reference Source

[ref-41] Topol J : Interview: Jáchym Topol.Interview by Kateřina Svobodová, translated from Czech by Klára Synková, edited by Iliya Ansky. *Prague Monitor*.2010a; Accessed 12 October 2023. Reference Source

[ref-71] Topol J : Gargling with Tar.Translated by David Short. Portobello Books,2010b. Reference Source

[ref-39] Topol J : The Devil's Workshop.Translated by Alex Zucker. Portobello Books,2013. Reference Source

[ref-40] Topol J Zucker A : Jáchym Topol & Alex Zucker | Interview | A sensitive person. *YouTube,*uploaded by Czech Centre London.2023; Accessed 12 October 2023. Reference Source

[ref-42] Vadde A Majumdar S : Introduction: criticism for the whole person.In: *The Critic as Amateur*. Edited by Saikat Majumdar and Aarthi Vadde, Bloomsbury Publishing,2020;1–28. Reference Source

[ref-43] Vane K : Kate reviews: The Devil's Workshop. *TNBBC*.2015; Accessed 12 October 2023. Reference Source

[ref-44] Vardoulakis D : Freedom from the free will: on kafka's laughter.SUNY Press,2016. Reference Source

[ref-45] Woods M : Translating Topol: Kafka, the Holocaust, and humor. *Slovo a smysl-Word and Sense.* Praha,2016;25(13):83–97. Reference Source

