# Supplementary material for: Lost in translation? Jáchym Topol’s The Devil’s Workshop and its local and transnational reader
Source: Open Res Eur. 2025 Feb 12;4:185. Originally published 2024 Aug 16. [Version 2] doi: 10.12688/openreseurope.17816.2 (PMC11842957; doi:10.12688/openreseurope.17816.2)
Supplement: Peer review history

# Reviewer report: Reviewer response for version 2

**DOI:** 10.21956/openreseurope.21095.r51141

**Published:** 2025-02-20

**Authors:** Yasser Ahmed Gomaa

**Affiliations:** 1 Assiut University, Asyut, Assiut Governorate, Egypt

The author has diligently addressed the reviewer feedback in her revised article. She provided a more detailed explanation of the methodology, including sampling methods and coding criteria, enhancing the transparency and credibility of the study. Additionally, she incorporated reader response theory to strengthen the theoretical foundation and ensure clearer links between reader reviews and the theoretical framework. Through meticulous revisions, the author improved the clarity and readability of the text by editing complex sentences and removing redundant discussions. While she chose not to add comparative analysis, her focused approach effectively highlights how Topol’s unique literary style and use of dark humor provoke thought and self-reflection on WWII memory and heritage. The revised version of the article meets the reviewers' suggestions and presents a more robust and comprehensive analysis.

 I recommend accepting the revised version.

If any, are all the source data and materials underlying the results available?

Partly

Is the work original in terms of material and argument?

Partly

Is the work clearly and cogently presented?

Partly

Is the argument persuasive and supported by evidence?

Partly

Does the research article contribute to the cultural, historical, social understanding of the field?

Yes

Does it sufficiently engage with relevant methodologies and secondary literature on the topic?

Partly

Reviewer Expertise:

Linguistics and Translation Studies.

I confirm that I have read this submission and believe that I have an appropriate level of expertise to confirm that it is of an acceptable scientific standard.

# Reviewer report: Reviewer response for version 1

**DOI:** 10.21956/openreseurope.19260.r45455

**Published:** 2024-11-08

**Authors:** Mustafa Kirca

**Affiliations:** 1 Çankaya University, Ankara, Turkey

In this article exploring Czech writer and poet Jáchym Topol’s writing style and particularly the reception of the novel *The Devil’s Workshop*, it is shown that despite being written primarily for Czech readers (and Slovak readers to some extent due to the close historical, lingual, and cultural proximity of the two nations), Topol’s novel under scrutiny has earned worldwide acclamation. I would like to read more about reception studies. Elaborating more on the theoretical background of the research and the role that translation plays in world literature and transnational reception, the paper can closely engage with relevant methodologies and secondary literature on the topic.

If any, are all the source data and materials underlying the results available?

Yes

Is the work original in terms of material and argument?

Yes

Is the work clearly and cogently presented?

Yes

Is the argument persuasive and supported by evidence?

Yes

Does the research article contribute to the cultural, historical, social understanding of the field?

Yes

Does it sufficiently engage with relevant methodologies and secondary literature on the topic?

Partly

Reviewer Expertise:

Comparative Literature and Translation Studies

I confirm that I have read this submission and believe that I have an appropriate level of expertise to confirm that it is of an acceptable scientific standard.

## References

[1] : The making of world literature: Turkish fiction as a case study. *Neohelicon* .2019;46(2) : 10.1007/s11059-019-00502-4 411-433 10.1007/s11059-019-00502-4

# Reviewer report: Reviewer response for version 1

**DOI:** 10.21956/openreseurope.19260.r43869

**Published:** 2024-10-04

**Authors:** Yasser Ahmed Gomaa

**Affiliations:** 1 Assiut University, Asyut, Assiut Governorate, Egypt

Report on the Article: "Lost in Translation? Jáchym Topol’s *The Devil’s Workshop* and its Local and Transnational Reader"

  

- This article, written by Bernadette Ščasná, examines Jáchym Topol's novel “The Devil’s Workshop,” focusing on its reception among both local (Czech) and international readers.
- The article situates the novel within the framework of transnational literature and memory studies, exploring how Topol’s unique writing style and use of dark humor navigate difficult themes such as Holocaust memorialization, genocide tourism, and memory entrepreneurship.
- The research employs a reception study methodology by analyzing online reviews of the novel from platforms like Amazon and Goodreads to understand how different audiences interpret the work’s treatment of World War II and the Holocaust, particularly in the contexts of Czech and Belarusian historical memory.
- The focus on Jáchym Topol's novel and its transnational reception provides an original contribution to the study of Czech literature in the context of global memory debates. The analysis of how the novel fits into the realm of transnational literature and the juxtaposition of Czech and Belarusian World War II narratives is a unique angle.
- The article relies heavily on established concepts such as difficult heritage and genocide tourism, which have been well-explored in memory studies. While the application of these concepts to The Devil’s Workshop is valuable, the novelty lies more in the case study (Topol's novel) than in the theoretical framework itself.
- To improve originality, the author could introduce new insights or comparative frameworks that extend beyond Topol’s novel, such as contrasting it with other works of transnational literature or examining how similar narratives have been treated in non-European contexts. Expanding on the novel’s specific contribution to transnational literature, beyond its local context, would also enhance originality.
- The article incorporates established theories from memory studies (e.g., Jan Assmann’s (ref 1)work on communicative and cultural memory, and Alison Landsberg’s work on prosthetic memory) to frame the novel's treatment of World War II narratives. It also uses reader reception as a methodological approach by analyzing online reviews of the novel.
- The analysis of reader reviews is innovative but lacks detailed methodological explanation. The article does not clearly describe how the reviews were selected, coded, or analyzed. Additionally, while the secondary literature on memory studies is strong, more engagement with literary reception theory would strengthen the analysis of how international readers process Topol’s work.
- The author should provide a more detailed methodology section, explaining how the reviews were gathered, how they were qualitatively analyzed, and what criteria were used for coding the data. This would enhance the transparency and rigor of the study. Engaging with additional literature on literary reception could also deepen the theoretical analysis of how transnational audiences engage with culturally specific texts.
- The structure of the article is generally clear, with a logical progression from the introduction, through the analysis, to the conclusion. The conceptual framework is well-defined, and the use of examples from the novel is effective in illustrating the key arguments.
- There is some repetition of ideas, particularly concerning the concepts of difficult heritage and memory tourism, which are discussed multiple times without adding new depth. Some sentences are overly complex and could be simplified for better readability. Additionally, the analysis sometimes veers too much into theory without linking it back clearly to the novel or the reader reviews.
- The author should revise sections where ideas are repeated to improve the flow of the text. Simplifying complex sentences will enhance clarity and readability. More explicit connections between the theoretical discussions and the practical analysis of the novel’s reception would strengthen the overall coherence of the argument.
- The argument about how The Devil’s Workshop navigates the intersection of local and transnational memory is well-supported by examples from the novel, particularly its treatment of Terezín and Khatyn as sites of memory. The use of reader reviews as evidence of how different audiences interpret the novel is a strong point.
- The connection between the reader reviews and the theoretical claims is not always fully developed. While reviews are cited, there is limited discussion of how these reviews support the argument that local and transnational readers engage with the novel differently. Additionally, some claims about transnational memory are not sufficiently backed by specific evidence from the reviews or the text.
- The author should expand the analysis of the reviews, providing more specific examples and linking them directly to the claims being made about local and transnational reception. This would make the argument more persuasive and grounded in concrete evidence.
- The article provides information about the sources of the reviews (Amazon, Goodreads, and Databazeknih.cz) and explains that the reviews were manually coded and analyzed.
- The actual review data are not made available, nor is there a detailed explanation of how the reviews were selected or coded. This limits the transparency of the study’s methodology.
- To address this issue, the author should either make the dataset of reviews available (if privacy concerns permit) or provide a detailed appendix explaining the coding process and offering sample reviews to illustrate how the analysis was conducted. This would enhance the scientific rigor of the article.
- The article contributes meaningfully to the field of memory studies, particularly by exploring how literature can function as a tool for reflecting on cultural memory, dark heritage, and the ethical challenges of memorialization in a transnational context. It also adds to the understanding of how Czech literature interacts with global historical narratives, particularly in relation to the Holocaust and World War II. The exploration of transnational memory and how different cultural groups interpret memorialization contributes to the broader discourse on how traumatic historical events are remembered and commodified in different societies.
- To ensure the article meets the highest academic standards, the author should address the following key areas:
  1. While the article offers a unique case study, it should delve deeper into the novel contributions of the study, such as how Topol’s work differs from other literary works that engage with difficult heritage. Comparative analysis with similar works could also enhance originality.
  2. The article needs a more detailed explanation of how the reader reviews were selected, coded, and analyzed. Offering transparency in the methodological process (e.g., coding criteria, sampling methods) is essential for scientific rigor.
  3. Revising complex sentences and removing redundant theoretical discussions will improve the flow and clarity. Streamlining the argument and ensuring that each section adds a new dimension to the analysis will also enhance readability.
  4. The author should provide more detailed examples of how the reader reviews directly support the claims being made about transnational memory. A clearer link between the reviews and the theoretical framework would make the argument more convincing.
  5. The source data (i.e., reviews) should be either made publicly available or summarized in an appendix with more detail about how they were analyzed. This would improve the study’s transparency and credibility.
- By addressing these points, the article will be more scientifically sound, making a stronger contribution to the fields of memory studies, literary reception, and transnational literature.

If any, are all the source data and materials underlying the results available?

Partly

Is the work original in terms of material and argument?

Partly

Is the work clearly and cogently presented?

Partly

Is the argument persuasive and supported by evidence?

Partly

Does the research article contribute to the cultural, historical, social understanding of the field?

Yes

Does it sufficiently engage with relevant methodologies and secondary literature on the topic?

Partly

Reviewer Expertise:

Linguistics and Translation Studies.

I confirm that I have read this submission and believe that I have an appropriate level of expertise to confirm that it is of an acceptable scientific standard, however I have significant reservations, as outlined above.

## References

[1] : Cultural memory and early civilization: Writing, remembrance, and political imagination. Cambridge University Press.2011;

[2] : “Difficult heritage” in nation building: South Korea and Post-Conflict Japanese colonial occupation architecture. Springer. 2019;

[3] : The Palgrave Handbook of Dark Tourism Studies. Springer. 2018;

[4] : Prosthetic memory: The transformation of American remembrance in the age of mass culture. Columbia University Press.2004;

[5] : Difficult heritage: Negotiating the Nazi past in Nuremberg and beyond. Routledge.2010;

# Response

**Published:** 2025-01-22

**Authors:** Bernadette Ščasná

**Affiliations:** Tallinn University, Tallinn, Harju County, Estonia

First, I would like to thank the reviewers for the time they dedicated to reading my article and providing feedback. I will now provide a description of the changes based on the comments I have received:

**Comment: **The article needs a more detailed explanation of how the reader reviews were selected, coded, and analyzed. Offering transparency in the methodological process (e.g., coding criteria, sampling methods) is essential for scientific rigor. The source data (i.e., reviews) should be either made publicly available or summarized in an appendix with more detail about how they were analyzed. This would improve the study’s transparency and credibility.

**Response:** I have added a more detailed explanation of the methodology in the Notes section where I describe the sampling methods, coding of the reviews etc. The reviewer also asked to make the reviews available or to provide more details on how they were analyzed. The datasets will not be made publicly available, however, the description of the methodology explains in more detail how the analysis and coding was done. Furthermore, I have added a note explaining that all the reviews are freely accessible on the respective platforms.  

**Comment:** The author should provide more detailed examples of how the reader reviews directly support the claims being made about transnational memory. A clearer link between the reviews and the theoretical framework would make the argument more convincing. Additionally, while the secondary literature on memory studies is strong, more engagement with literary reception theory would strengthen the analysis of how international readers process Topol’s work.

**Response:** I have added sections on reader response theory in the introduction order to strengthen the theoretical foundation of the work as suggested by the reviewer. The reader response theory is also applied in the analysis. This also enhanced the link between the theoretical framework and the reviews. Furthermore, all the claims have been revised to ensure they are all sufficiently supported.  

**Comment:** Revising complex sentences and removing redundant theoretical discussions will improve the flow and clarity. Streamlining the argument and ensuring that each section adds a new dimension to the analysis will also enhance readability.

**Response:** I have edited complex sentences and removed repetitions to improve the text's clarity.  

**Comment:** While the article offers a unique case study, it should delve deeper into the novel contributions of the study, such as how Topol’s work differs from other literary works that engage with difficult heritage. Comparative analysis with similar works could also enhance originality.

**Response: **I decided not to address this point, as my work was not intended to be comparative. It rather focuses on showcasing how Topol’s particular writing style and use of different literary devices and aesthetics can affect the readers in terms of provoking thoughts related to important issues related to WWII memory and its difficult heritage.
